# Glycine receptors and brain development

**DOI:** 10.3389/fncel.2013.00184

**Published:** 2013-10-21

**Authors:** Ariel Avila, Laurent Nguyen, Jean-Michel Rigo

**Affiliations:** ^1^Cell Physiology, BIOMED Research Institute, Hasselt UniversityDiepenbeek, Belgium; ^2^Groupe Interdisciplinaire Génoprotéomique Appliquée-Neurosciences, Centre Hospitalier Universitaire Sart Tilman, University of LiégeLiège, Belgium; ^3^Groupe Interdisciplinaire Génoprotéomique Appliquée-Research, Centre Hospitalier Universitaire Sart Tilman, University of LiègeLiège, Belgium; ^4^Walloon Excellence in Life Sciences and Biotechnology, Centre Hospitalier Universitaire Sart Tilman, University of LiègeLiège, Belgium

**Keywords:** glycine receptors, GlyRs, glycine, brain development, cortex, hippocampus, interneurons, migration

## Abstract

Glycine receptors (GlyRs) are ligand-gated chloride ion channels that mediate fast inhibitory neurotransmission in the spinal cord and the brainstem. There, they are mainly involved in motor control and pain perception in the adult. However, these receptors are also expressed in upper regions of the central nervous system, where they participate in different processes including synaptic neurotransmission. Moreover, GlyRs are present since early stages of brain development and might influence this process. Here, we discuss the current state of the art regarding GlyRs during embryonic and postnatal brain development in light of recent findings about the cellular and molecular mechanisms that control brain development.

## INTRODUCTION

Glycine receptors (GlyRs) are mainly known by their function in spinal circuits. They are widely expressed in the spinal cord where they contribute to synaptic transmission (Colin et al., 1998; Moss and Smart, 2001; Scain et al., 2010), and play an important role in motor control (Rees et al., 2003), and pain perception (Harvey et al., 2004; Zeilhofer, 2005; Lynch and Callister, 2006). They appear early during spinal cord development and their subunit composition is developmentally regulated (Watanabe and Akagi, 1995). Interestingly, it has been shown that GlyRs influence spinal development by affecting interneuron differentiation (McDearmid et al., 2006) and synaptogenesis (Scain et al., 2010). Although the occurrence of functional GlyRs in the adult brain was already described two decades ago (Malosio et al., 1991b; Chattipakorn and McMahon, 2002), little is known regarding their function in supratentorial regions. It is believed that GlyRs mainly mediate extra-synaptic tonic inhibition in the hippocampus (Song et al., 2006; Keck et al., 2008; Zhang et al., 2008; Xu and Gong, 2010; Aroeira et al., 2011). Moreover, synaptic GlyRs have also been identified in the brain cortex (Waseem and Fedorovich, 2010), excitatory, and inhibitory hippocampal neurons (Brackmann et al., 2004; Danglot et al., 2004; Kubota et al., 2010; Xu and Gong, 2010), the cerebellum (Pedroarena and Kamphausen, 2008), and thalamic (Ghavanini et al., 2005) and brainstem nuclei (Turecek and Trussell, 2001). In addition, disruption of GlyRs activity contributes to brain pathologies. Alternative splicing variants have been detected in patients suffering from temporal lobe epilepsy (Eichler et al., 2009). Moreover, mutations in genes encoding either the GlyR alpha 2 subunit (Piton et al., 2011) or the aminomethyltransferase enzyme (AMT), which is an important factor involved in the degradation of glycine (Yu et al., 2013), have been found in patients affected by autism.

GlyRs are trans-membrane protein complexes formed by the assembling of five subunits arranged symmetrically around a central pore. Five types of GlyR subunits, four alphas and one beta, have been described so far (Matzenbach et al., 1994; Lynch, 2009). Moreover, alternative splicing can generate additional variants for some alpha subunits (Kuhse et al., 1991; Malosio et al., 1991a; Nikolic et al., 1998; Legendre et al., 2009). Alpha subunits can form homo- or heteromeric receptors in combination with the beta subunit (Pribilla et al., 1992; Oertel et al., 2007). The alpha one/beta combination displays the fastest kinetic and has been associated with mature synapses mediating fast inhibitory neurotransmission, mainly in the spinal cord and in the brainstem. In addition, the beta subunit is the only one able to interact with gephyrin and, hence, provides an anchoring mechanism necessary for synaptic localization and function (Kirsch et al., 1995). Complementarily, alpha two containing receptors, which display slower kinetics characterized by a slower desensitization, are abundant during development and have been found in extra-synaptic locations. Besides glycine, GlyRs can also be activated by other ligands, such as taurine and alanine (Schmieden et al., 1992; Kilb et al., 2008). Each of these molecules can bind to the extracellular domain and promote the opening of the central pore that allows the selective flux of chloride and bicarbonate ions. In the same way as for type A gamma-aminobutyric acid receptors (GABA_A_R), GlyR-mediated anion flux is solely dependent on the electrochemical gradient established across the cell membrane for these ions.

During development, chloride gradients evolve in function of the expression of chloride transporters. In neurons, the potassium-chloride cotransporter 2 (KCC2) actively reduces the intracellular concentration of chloride, transforming the opening of a chloride channel into a hyperpolarizing stimulus (Lee et al., 2005; Zhu et al., 2005). Conversely, the sodium-potassium-chloride co-transporter (NKCC1) exerts the opposite effect and thus increases the intracellular concentration of chloride transforming the ion channel-mediated chloride flux into a depolarizing signal. In consequence, GlyR activation during embryonic and early postnatal development most likely induces a depolarization of the cell membrane (Flint et al., 1998; Kilb et al., 2002, 2008) which in turn may activate calcium influx (Platel et al., 2005; Young-Pearse et al., 2006). Compared to glutamate-mediated depolarization, chloride efflux is thought to be less harmful to the cell since there is no risk to induce excitotoxicity. This is a remarkable feature shared with GABA_A_Rs-mediated signaling during development and has been associated with developmental functions.

Here, we review the accumulating literature regarding the role of GlyRs during embryonic and postnatal brain development. We discuss this literature in the context of recent findings about the cellular and molecular events that govern brain development and we provide insight into the processes in which these receptors may be involved.

## GLYCINE RECEPTORS DURING EMBRYONIC BRAIN DEVELOPMENT

Brain development is a long process that starts early during embryogenesis and takes years to be completed in humans. The initial steps involve precise coordination of cell proliferation, differentiation, and cell migration. Tight control of these processes is achieved by integration of the intrinsic genetic program with the extracellular signals present in the biochemical environment (Pla et al., 2006; Caronia-Brown and Grove, 2011). Interestingly, neurotransmitters and their receptors are part of this biochemical environment and contribute to early steps of neurogenesis (Nguyen et al., 2001; Soria and Valdeolmillos, 2002; Heng et al., 2007). GlyR expression in the CNS was first studied using radioactive labeled strychnine; however, this approach only identified high affinity receptors, which are mostly absent during development (Frostholm and Rotter, 1985; Probst et al., 1986). To circumvent this problem, Malosio and colleagues used *in situ* hybridization to assess the presence of various GlyR subunits in the rat embryonic brain (Kuhse et al., 1991; Malosio et al., 1991b). In this study, the authors found abundant expression of both alpha 2 and beta subunits during prenatal stages. The messenger RNA (mRNA) for the alpha 2 subunits was the most widely expressed and it was detected since embryonic day 14 (E14) onwards in all layers of the cortex during the whole embryonic period (Malosio et al., 1991b). This mRNA was also found in the diencephalon and midbrain, at E14, and in the thalamus and cerebellum primordium, at E19. The beta subunit mRNA was found mainly in the cortex at E14, where it was distributed homogenously. However, at E19 the expression pattern became more restricted and only layers I and II were labeled. Interestingly, at E19 the cerebellum was also selectively labeled with the GlyR beta subunit-directed probes. No other GlyR subunits were detected in the brain, but it is important to note that the expression of the GlyR alpha 4 subunit has not yet been studied at this age. Additionally, at E19, immunostainings detected GlyR in radially oriented neurons in the intermediated zone (IZ) and cortical plate (CP; Flint et al., 1998). Recently, western blot analysis and immunolabeling have been used to demonstrate the presence of GlyR alpha 2 subunit in migratory interneurons isolated from the brain cortex at the E13 (Avila et al., 2013). Protein expression was found on progenitors as well as on migrating neurons at this age. This was a first step to further study the involvement of GlyRs on interneuron development (see below).

Contrasting with the accumulating evidence regarding GlyR expression during cortical development, there is only limited information about the functionality and physiological relevance of these receptors in the embryonic brain. To examine when functional GlyRs first appear during cortical neurogenesis, Flint et al. (1998) recorded glycine and taurine elicited currents in different zones of the embryonic cortex at E19. Interestingly, all recorded neurons in the CP and IZ showed GlyR-mediated responses. In contrast, none of the cells located in the ventricular zone (VZ) seemed to have functional GlyRs (Flint et al., 1998). These results were confirmed by immunohistochemical analyses, which showed intense labeling in the IZ and CP, but not in the VZ. Single cell resolution was achieved in the IZ where GlyR expression was localized in the soma and apical leading process (Flint et al., 1998). Complementarily, *in vitro* experiments, carried out at E17 in rats, demonstrated that newborn projection neurons already express GlyRs, along with GABA_A_Rs and voltage-gated ion channels at this age, by the end of their radial migration (Noctor et al., 2004). Calcium measurements have provided further evidence about the presence and function of GlyRs in the embryonic cortex. Consistent with a depolarizing effect, it was shown that glycine application triggers a massive calcium influx in the upper-layer pyramidal neurons at E17. This effect was blocked by strychnine and totally absent in the GlyR-knockout (KO) animals (Young-Pearse et al., 2006). More interestingly, the same effect was recorded very early during cortex development, at E13 (Platel et al., 2005). At this age, GlyRs were shown to have a unique role in preplate (PP) neurons, which are depolarized in response to GlyR activation. This depolarization activates voltage-sensitive sodium channels that subsequently activate sodium-sensitive calcium transporters. This complex channel association leads to the rise in intracellular calcium and promotes vesicular release of glutamate (Platel et al., 2005). Importantly, this role was exclusively mediated by GlyRs and did not involve the activation of co-expressed GABA_A_Rs. Moreover, released glutamate amplified GlyR-mediated signaling, and triggered calcium influx in the VZ as well (Platel et al., 2005). Complementarily, it has recently been shown that differentiated human midbrain-derived cells express functional GlyRs that respond to glycine with low affinity, whose activation can trigger calcium influxes (Wegner et al., 2012). These cells were obtained from 10 to 16 weeks human fetuses and kept in culture for 1–3 weeks. After that period, they expressed GlyRs, but they had limited impact on neurogenic capability and cell differentiation towards dopaminergic neurons (Wegner et al., 2012). Thus, the impact of GlyRs activation on cell differentiation remains poorly understood.

### GLYCINE RECEPTORS AND CELL MIGRATION IN THE DEVELOPING BRAIN

Recently, we have gained a better understanding of the mechanisms that control this process and the role of extracellular factors involved. Interestingly, calcium-dependent processes seem to be fundamental for transducing molecular signalings associated with activation of neurotransmitter receptors. Indeed, it has been demonstrated that the secondary activation of voltage-gated calcium channels (VGCCs) is necessary to mediate GABA actions on cell migration (Bortone and Polleux, 2009) and proliferation (LoTurco et al., 1995; Owens et al., 1996). Along this line, a short report has suggested that GlyR could have an influence in radial migration during late embryonic development (Nimmervoll et al., 2011). Application of glycine was shown to impede radial migration in agreement with the effect caused by the activation of type C GABA receptors (GABA_C_Rs; Denter et al., 2010), but opposed to the effects of GABA acting at GABA_A_R activation that rather promotes radial migration (Manent et al., 2005). As an explanation to the opposite effects exerted by the activation of GABA receptors it has been proposed that GABA_C_Rs could exert their effect by an ion-independent process (Denter et al., 2010). This could be a likely hypothesis to explain migration arrest caused by GlyRs activation. Moreover, GlyRs could also act using non-autonomous mechanisms. As it has been presented, GlyRs are distributed throughout the whole cortex at this age and they may contribute to different processes to indirectly control cell migration. Nevertheless, the role of GlyRs in controlling radial cell migration remains to be demonstrated *in vivo*. On the other hand, the role of GlyRs in controlling interneurons migration has recently become clearer. Specifically, it has been demonstrated that GlyR activation can trigger a series of intracellular events in cortical interneurons that leads to the modulation of interneuron migration during embryonic development (Avila et al., 2013). Molecular events triggered by GlyR activation include the secondary activation of VGCC that leads to the influx of calcium and to the modulation of spontaneous oscillations. Calcium influx, mediated by the activation of L-type channels, indirectly leads to the modulation of myosin activity and to changes in actomyosin contractions at the rear of the migratory neurons. This further modifies interneuron nucleokinesis and affects interneuron migration speed (Avila et al., 2013). These findings provide evidence for a role of GlyRs in interneuron development and link the effect of a neurotransmitter receptor to the remodeling of the cytoskeleton.

Strikingly, it has been reported that GlyR alpha 2 KO animals (GlyRa2^-^/^-^) do not display any evident morphological disruption of the cortex at postnatal day 0 (P0; Young-Pearse et al., 2006). Moreover, in these animals, Notch1, Id2, Btg2, TUG1, and GABA_A_R subunit 6 expression appeared to be completely normal around birth, suggesting that GlyR alpha 2 subunit was not required for the acquisition of the main morphological and biochemical features of the cortex (Young-Pearse et al., 2006). The absence of morphological defects was suspected to arise from compensatory up-regulation of other GlyR or GABA_A_R subunits in GlyRa2^-^/^-^ animals. In this regard, it is interesting to note that GlyRa2^-^/^-^ animals displayed glycine elicited responses at P7 (Young-Pearse et al., 2006), suggesting that other GlyR subunits could still be necessary for postnatal development. A possible candidate is the GlyR alpha 3 gene, which has been found selectively expressed in layer 2/3 of the adult cortex (Sorensen et al., 2013). Recently, re-analysis of a new GlyRa2^-^/^-^ mouse line has revealed a marked dysfunction in interneuron development. GlyR alpha2 deficient mice were found to have fewer cortical interneurons that migrated at a lower speed while navigating the sub-ventricular zone (SVZ) at the E15 (Avila et al., 2013). This defect was previously unnoticed, most probably due to the methodology applied in the early studies. Interneurons are about 15% of the total number of neurons in the cortex (Sahara et al., 2012) and even a marked reduction in their number is likely to be unnoticed using classical histological methods. Nevertheless, cortical interneurons play an active role controlling cortical excitability (Seybold et al., 2012) and have been proposed to be fundamental in starting immature circuits (Ben-Ari et al., 2004). Moreover, their dysfunction has been associated with the development of different brain pathologies (Cobos et al., 2005; Gogolla et al., 2009; Chattopadhyaya and Cristo, 2012; Marin, 2012).

Apart from its involvement in the control of cell migration, GABA has been involved in the control of cell proliferation as well (Represa and Ben-Ari, 2005). In this regard, actions of glycine or taurine are much less explored, but it has been shown that certain brain neuronal progenitors also express GlyRs (Nguyen et al., 2002). Thus, glycine or taurine could also contribute to the neurogenesis process in the brain. Indeed, knockdown of GlyRs in the spinal cord induced an increase in the number of mitotic cells (McDearmid et al., 2006) arguing in favor of a role of glycine in cell cycle regulation.

## GLYCINE RECEPTORS DURING POSTNATAL BRAIN DEVELOPMENT

After birth, cell proliferation is restricted to small areas in the brain while intense neuronal migration stops to allow final cell positioning, morphological differentiation, and synaptogenesis. At this age, GlyR alpha 2 and beta transcripts are abundant in the cortex and other brain structures (Malosio et al., 1991b; Okabe et al., 2004). However, there is a dynamic change in levels of expression of these mRNAs during the first two postnatal weeks of development. At the first postnatal day (P0), the alpha 2 and beta transcripts can be detected throughout the whole cortex, the thalamus, and the hippocampus (Kuhse et al., 1991; Malosio et al., 1991b). Complementary studies have found a homogeneous expression of alpha 2 and beta subunits in CP and Cajal–Retzius cells at this age (Okabe et al., 2004). Later during development, at P5, alpha 2 subunits have been detected specifically in layers I/II and IV while the beta mRNA probe displayed a preferential labeling of layers I/II and VI in the cortex. Moreover, the evidence suggests that this pattern keeps on changing during the next 10 days and by P15 it reaches what resembles the adult distribution. At this age, while the alpha 2 transcripts display strong labeling in layer VI, the beta transcripts are detected in all the layers of the cortex, the hippocampus and the cerebellum (Malosio et al., 1991b). In addition, the transcripts for the alpha 1 and alpha 3 subunits are also detected in the brain after the second postnatal week of development (Malosio et al., 1991b; Sorensen et al., 2013). With respect to the drastic decrease of GlyR alpha 2 subunit in the brain it has been suggested that alpha 2 subunit containing receptors could gradually been replaced by alpha 1/beta heteromers, similarly to what happens in the spinal cord. However, recent analyses, at the protein level have shown that the increase in alpha 1 subunit is so small that it could hardly replace the expression of the alpha 2 subunit, which seems to minimally decrease after birth (Jonsson et al., 2012). This is in line with functional studies that support the expression of homomeric alpha 2 containing receptors in the adult hippocampus (Chattipakorn and McMahon, 2002).

### CORTICAL GLYCINE RECEPTORS

Several electrophysiological experiments have explored the functionality and pharmacological properties of GlyRs in the postnatal cortex (Flint et al., 1998; Kunz et al., 2012). Specifically, by using patch clamp experiments, it has been shown that both Cajal–Retzius cells and CP neurons express functional GlyRs. Interestingly, GlyR-elicited currents were three times larger in Cajal–Retzius cells compared to CP neurons at the same age (Okabe et al., 2004). Despite the evident difference, there were no other pharmacological or molecular differences in terms of GlyR response or subunit composition, which suggested that GlyRs in both cell types may consist of alpha 2/beta heteromeric receptors at least during the first postnatal days (Okabe et al., 2004). Functional GlyRs, with similar subunit composition, have also been described in subplate (SP) neurons, where cells respond with less affinity to taurine compared to glycine and beta-alanine in the same way as it has been shown for CP and Cajal–Retzius cells (Kilb et al., 2008). Activation of GlyRs in the postnatal brain can cause different biological effects depending on the cell type. During early postnatal ages, application of glycine to CP neurons in voltage-clamp mode induces a sustained current along with intense postsynaptic current events (Flint et al., 1998). This neurotransmitter release facilitation seems to persist in the developing visual cortex even after the third postnatal week (Kunz et al., 2012). In contrast to these studies, the application of glycine to Cajal–Retzius cells induces a shunting inhibition of evoked action potentials, hence blocking synaptic transmission (Kilb et al., 2002). A key factor during the establishment of the first synapses in the cortex is the arrival of extra cortical inputs carried by thalamo-cortical axons. These axons primarily innervate SP neurons, which subsequently transfer the information to the rest of the layers in the cortex. It has been shown that GlyRs present in those cells display properties similar to those of receptors present in the CP, and upon activation they depolarize the cells lowering the threshold for the generation of action potentials (Kilb et al., 2008). An interesting possibility that arises from this study is that the depolarization induced by GlyRs activation can subsequently also modulate neuronal activity in downstream cortical networks (Dupont et al., 2006) and influence their development.

### HIPPOCAMPAL GLYCINE RECEPTORS

Functional GlyRs are also found in other regions of the brain. In the developing hippocampus, GlyRs have been reported to be composed by alpha 2/beta heteromeric and alpha 2 homomeric receptors (Thio et al., 2003). Interestingly, they have been found on pyramidal neurons from the regions CA1 and CA3 (Ito and Cherubini, 1991; Keck et al., 2008) and also on Mossy fibers boutons (Kubota et al., 2010). Physiological consequences of GlyRs activation have been demonstrated to be developmentally regulated and to contribute sequentially to depolarization and hyperpolarization in the CA3 region (Ito and Cherubini, 1991). This depolarization effect converts into hyperpolarizing around P7 and occurred at the same time of the shift in the action of GABA in agreement with the similar chloride conductance characteristic of GABA and glycine ionotropic receptors (Ito and Cherubini, 1991). Despite of that, depolarizing actions of glycine through the activation of GlyRs can be found later in the CA1 region of the hippocampus during and after the third postnatal week (Song et al., 2006; Chen et al., 2011). In fact, the postnatal inhibitory shift depends on the region under investigation (see below). The consequences of the early depolarization exerted by GlyRs are not clear, but they include modulation of receptor trafficking at the synapse (Chen et al., 2011) and facilitation of neurotransmitter release. Moreover, extrasynaptic GlyRs contribute to membrane depolarization, tonic inhibition, and long-term depression in the CA1 region (Song et al., 2006; Keck et al., 2008; Xu and Gong, 2010; Chen et al., 2011). Furthermore, GlyRs have been found on Mossy fiber boutons (Kubota et al., 2010) and hilar synaptic terminals, where they enhance excitatory activity (Lee et al., 2009).

### CEREBELLAR GLYCINE RECEPTORS

In the cerebellum, GlyRs have been found functionally expressed on Golgi (Dieudonne, 1995), granular (Kaneda et al., 1995), and Purkinje cells during the first two postnatal weeks (Kawa, 2003a). Additionally, they have also been found in the deep cerebellar nuclei, where they contribute to synaptic (Pedroarena and Kamphausen, 2008) and extra synaptic (Kawa, 2003b) transmission. Interestingly, pharmacological experiments suggest the involvement of alpha 2 homomeric receptors in cerebellar synapses at P7–P10 (Pedroarena and Kamphausen, 2008). This is particularly interesting since it is assumed that synaptic GlyRs are heteromeric receptors (Kirsch et al., 1995). As it has been described in other brain structures, GlyR activation increases the frequency of excitatory and inhibitory postsynaptic currents facilitating neurotransmitter release in the cerebellum (Kawa, 2003a). These effects were developmentally regulated and peaked at P5 for EPSCs and at P9 for IPSCs (Kawa, 2003a). Moreover, these effects were sensitive to TTX, suggesting the involvement of actions potentials and upstream activation of granular cells. This is supported by a previous study that has documented the presence of GlyRs in extra synaptic locations on granular cells (Kaneda et al., 1995). Synaptic GlyRs have also been found on Golgi cells early during development (Dieudonne, 1995). However, their glycinergic inputs and the consequences of GlyR activation for the cerebellar network are currently unknown.

### THALAMIC GLYCINE RECEPTORS

In the thalamus, despite the clear labeling of this structure by GlyR probes (Kuhse et al., 1991; Malosio et al., 1991b), their physiological function has only recently been studied. Remarkably, GlyRs have been found to be involved in more than half of the inhibitory synapses recorded in the ventrobasal thalamic nuclei. This function was observed at P13 in the rat brain and, although there is no certainty regarding any developmental function, it highlights the contribution of GlyRs to thalamic inhibition during development (Ghavanini et al., 2005).

### NETWORK FORMATION AND GLYCINE RECEPTORS

The development of the spinal cord and brainstem provides an intriguing example that involves GlyRs in circuit development. There, synaptic transmission changes from being predominantly GABAergic to glycinergic (Kotak et al., 1998; Gao et al., 2001; Rahman et al., 2013). This change consists of a presynaptic modification (Nabekura et al., 2004; Muller et al., 2006), but it also includes the replacement of alpha 2 with alpha 1 containing GlyRs. Transient expression of GABAergic neurotransmission has been extensively studied in the spinal cord (Gao et al., 2001) and it raises the question whether this is a more general phenomenon that can also occur in the brain. Indeed, GlyRs have been found co-localizing with GABAergic terminals in the hippocampus (Brackmann et al., 2004; Danglot et al., 2004) and in the primate cerebellum (Crook et al., 2006). Moreover, functional mixed synapses have been recorded in this brain region (Dumoulin et al., 2001; Dugue et al., 2005). Thus, since mixed synapses may be found during the transition between GABAergic to glycinergic transmission, future research aimed to understand the development of glycinergic synapses should clarify if this transition also occurs in the brain. Nevertheless, mixed synapses may contribute to the development and plasticity of the brain by affecting the functioning of specific circuits as it has been shown in the cerebellum (Dumoulin et al., 2001; Dugue et al., 2005). It is believed that GABAergic activity in mixed developing glutamatergic terminals promotes the development of excitatory connections (Gutierrez, 2003). Consequently, the presence of mixed synapses during development could be an effective mean to support the development of fast glycinergic neurotransmission. This could be particularly relevant if the developmental functions of GABA are exclusive and cannot be taken over by the activation of GlyRs. This is likely to be the case since it has been shown that GABAergic neurotransmission does not compensate for impaired glycinergic signaling at synaptic sites (Muller et al., 2008). On the other hand, GABAergic transmission can modify the function of glycine and GlyRs at mixed synaptic sites and provide specialized mechanisms to control excitability. In line with this, it has been shown that GABA can modulate GlyRs activation in the developing brain cortex (Breustedt et al., 2004). This modulation was only in one direction and while glycine-evoked currents were inhibited by GABA, GABA-evoked currents were only marginally affected by glycine. Similarly, cross inhibition of GABA_A_Rs and GlyRs have also been observed in the hippocampus, but occurring through a different mechanism involving the activation of both receptors (Li and Xu, 2002). Additionally, direct action of GABA on GlyRs should increase the complexity of glycinergic synapses in the brain (Lu et al., 2008; Singer, 2008).

The transition from slow GABAergic events to faster inhibitory glycinergic activity highlights the fact that network development may need transient, but sustained excitation. In addition, this can be enhanced by the slow decay time associated to certain GABA_A_Rs (Dunning et al., 1999) and to alpha 2 homomeric GlyRs, present in the immature brain. It is believed that the slow decay of GABA-elicited currents may contribute initially to achieve a long depolarization and to rise cytoplasmic calcium levels that in turn may promote dendrite and spine development (Marty et al., 1996; Konur and Ghosh, 2005) as well as synaptic stabilization and refinement (van den Pol, 2004). Indeed, it has been demonstrated that GABAergic depolarization precedes glutamatergic activity and promotes the establishment of synaptic circuits in some regions of the brain (Ben-Ari, 2002; Hennou et al., 2002; Gozlan and Ben-Ari, 2003; Ben-Ari et al., 2004). However, this is not a general rule as the opposite have been found in some regions of the cortex (Agmon and O’Dowd, 1992). Nevertheless, by homology to GABA_A_Rs, GlyRs may contribute to network formation in the regions where sustained depolarization is needed.

Finally, considering the important role of interneurons in the establishment of the first brain circuits (Cossart, 2011; Sauer and Bartos, 2011) and the recently described role of GlyRs in controlling interneuron development (Avila et al., 2013), it is tempting to speculate that GlyRs could indirectly modify early patterns of activity and have long lasting consequences in brain activity by affecting the number or positioning of cortical interneurons. GlyR knockout animals will potentially be very useful to understand the consequences of the involvement of GlyRs in circuit formation.

### DEPOLARIZING TO HYPERPOLARIZING SHIFT IN GlyRs-MEDIATED ACTIONS

Shift from depolarizing to hyperpolarizing action of glycine and GABA seems to be a general phenomenon and it has been shown to occur earlier in caudal than in more rostral parts of the CNS (Ehrlich et al., 1999; Ben-Ari et al., 2007). Indeed, this shift occurs around birth in the spinal cord (Wu et al., 1992), after P3 in the brainstem (Singer et al., 1998), after P7 in the cerebellum (Brickley et al., 1996), around P7–P12 in the CA3 region of the hippocampus (Ben-Ari et al., 1989; Ito and Cherubini, 1991; Tyzio et al., 2007) and around P16 in the cerebral cortex (Owens et al., 1996; for review see Ben-Ari et al., 2007). Moreover, depolarizing actions of glycine compared to GABA have been shown to occur at the same time in the spinal cord (Wu et al., 1992) brainstem (Backus et al., 1998; Singer et al., 1998), and the CA3 (Ito and Cherubini, 1991) and CA1 region of the hippocampus (Verheugen et al., 1999; Song et al., 2006; Chen et al., 2011). Thus, overall GlyRs seem to be part of the general developmental program of the brain complementing the role of GABA and providing flexibility and specialized mechanisms to control excitation since the early stages of brain development.

## GLYCINE RECEPTORS ENDOGENOUS AGONISTS DURING DEVELOPMENT

Endogenous activation of GlyRs in the developing cortex occurs through non-synaptic release of neurotransmitters and involves paracrine/autocrine mechanisms (Le-Corronc et al., 2011). Similar mechanisms operate for GABA and glutamate release contributing to analogous developmental functions (Manent et al., 2005; Manent and Represa, 2007). Similarities and differences between various neurotransmitters systems have been extensively covered by Le-Corronc et al. (2011). Until now, most of the developmental effects associated with glycine were only studied in the spinal cord, but as it has been discussed, glycine and GABA can serve to analogous functions in the developing brain. However, the effect of glycine has its own characteristics and it seems to be complementary to the effect exerted by the activation of GABA_A_Rs. Differences can be found in the control of interneuron migration. There, glycine directly controls interneuron motility (Avila et al., 2013) while GABA_A_Rs exclusively affect interneuron pausing time (Bortone and Polleux, 2009). Moreover, during radial migration, the activation of GABA_A_Rs has an opposite effect compared with the activation of GlyRs. More research is needed to explain these effects. Early studies suggested that taurine could be the endogenous ligand acting on immature GlyRs in the developing cortex. This suggestion was based on the presence of this amino acid in CP neurons (Flint et al., 1998), and on the detection of taurine and glycine in adult cortex (van den Pol and Gorcs, 1988). However, more recent studies have shown that levels of neurotransmitters can drastically change during the development of the brain and are essentially different from the concentrations found in the adult. While the levels of GABA in the parietal cortex of young adults are 2.6-fold higher than glycine, the levels of this amino acid are three- to fourfold lower than glycine in the embryonic brain. The concentration of taurine progressively increases during embryogenesis, reaching its maximum in the cortex around birth. Comparatively, E13 levels of taurine are 10- and 20-fold higher than the levels of glycine and GABA, respectively (Benitez-Diaz et al., 2003). However, it has been shown that cortical GlyRs (Kilb et al., 2002, 2008; Okabe et al., 2004), and specifically homomeric GlyRa2 (Schmieden et al., 1992; De Saint Jan et al., 2001) are 10 times less sensitive to taurine, which in addition, is only a partial agonist of GlyRs (Schmieden et al., 1992; Hussy et al., 1997; Kilb et al., 2002, 2008; Okabe et al., 2004). Moreover, it is worth noting that GlyRs affinity for their ligand can substantially increase due to RNA editing or splicing (Legendre et al., 2009). Altogether, this evidence suggests that both neurotransmitters, glycine and taurine, could act as ligands of GlyRs at various time points and locations during cortical development. In fact, we found that glycine is the main endogenous GlyRs agonist that promotes interneuron migration in the developing cortex and that taurine contributes to this process independently of GlyRs activation (Avila et al., 2013). In this study, glycine was found to be produced by immature projection neurons in the CP. Neuronal control of glycine has also been observed in the adult hippocampus (Yee et al., 2006), where it has been suggested that neurons could be a more important factor in the control of glycine compared to astrocytes, which have been classically involved in uptake of glycine. Despite of this, astrocytic release of glycine is important in the cortex and hippocampus, where it supports tonic inhibition by activation of GlyRs later during postnatal development (Zhang et al., 2008; Kunz et al., 2012). The extracellular level of glycine in the brain is controlled by the glycine transporter one (GlyT1), which is abundantly expressed (Cubelos et al., 2005).

Glycine transporters appear early during embryonic brain development in the rat (Jursky and Nelson, 1996). GlyT1 is predominant in the embryonic cortex and can be detected in radial glial cells (Jursky and Nelson, 1996). Although, to our knowledge, no developmental function has been given to this transporter at this age, its expression in radial glial cells suggest that these cells may play an important role by controlling the extracellular level of glycine through GlyT1. In fact, it has been proposed that this could be the primarily role of GlyT1 during spinal cord development where this transporter is instrumental in the removal of glycine from the extracellular compartment in extra synaptic locations (Gomeza et al., 2003). On the other hand, GlyT2 displays weak expression in the brain cortex and hippocampus, but is more abundant in the cerebellum (Jursky et al., 1994; Jursky and Nelson, 1996). Remarkably, the progressive development observed in the spinal cord where GlyT2 increases in expression to regulate the level of glycine in combination with GlyT1 (Lall et al., 2012) does not seem to occur in the developing brain. In contrast, later during postnatal development glycine level is exclusively controlled by GlyT1 independently of GlyT2 in the cortex (Gomeza et al., 2003; Kunz et al., 2012). Similar findings are observed in the hippocampus where the prominent function of GlyT1 found at P2 (Eulenburg et al., 2010) is also present in the adult formation (Martina et al., 2004; Yee et al., 2006). Nevertheless, GlyT2 is expressed by hippocampal interneurons and may contribute to additional functions (Danglot et al., 2004; Song et al., 2006).

During postnatal development, high performance liquid chromatography measurements have described another shift in the concentration of inhibitory neurotransmitters. According to these measurements, after a peak around birth, the levels of taurine remain high and rather constant during the first postnatal week. Then, taurine concentration decreases, but at P15 it still remains two and five times higher than GABA and glycine ones, respectively (Sturman, 1988; Benitez-Diaz et al., 2003). Functional experiments carried out at P6 demonstrate that taurine mainly activates glycine and not GABA receptors in the CP (Yoshida et al., 2004). The twofold increase in the level of glycine during embryogenesis reaches a peak around birth and gradually decreases during the first 2 weeks of postnatal development to about 60% of its original peak value. Interestingly, at this age certain components of the glycine cleavage system (GCS) are highly expressed in the cortex, the cerebellum, and the hippocampus (Ichinohe et al., 2004; Le-Corronc et al., 2011). This suggests that GABA and GlyRs could have an important role during a period of intense synaptogenesis. In addition, the fact that taurine levels remain above GABA and glycine even after the first 2 weeks of postnatal development, when GlyRs are activated almost exclusively by glycine (Yoshida et al., 2004), suggests that taurine could serve other functions independently of the sole activation of glycine or GABA receptors in the postnatal brain. Indeed, taurine is known to contribute to many different cellular functions in various physiological contexts, often by an unknown molecular mechanism (Ripps and Shen, 2012). Relevant targets for taurine during postnatal development may include KCC2, which is inhibited by taurine, preventing its earlier activation and the anticipated inversion of the chloride gradient (Inoue et al., 2012). Thus, once again, this evidence suggest that both neurotransmitters, glycine and taurine, could act as ligands of GlyRs depending on the time and place of occurrence during brain development. Furthermore, they could have differential effects at prenatal and postnatal ages.

## PATHOPHYSIOLOGICAL CONSEQUENCES OF GlyRs ACTIVATION DURING DEVELOPMENT

While the identity of the endogenous GlyRs ligands remains unclear, both glycine and taurine seem to have an important role during brain development. The concentration of glycine is influenced by the GCS that catalyzes the degradation of glycine and provides the developing brain with other metabolites, such as 5,10-methylenetetrahydrofolate, which is essential for DNA synthesis. Failure in GCS activity leads to serious malformations, such as agenesis of the corpus callosum, gyral malformation and cerebellar hypoplasia. Interestingly, mutations of the AMT gene, encoding an enzyme essential for the degradation of glycine, have been found in two autistic patients (Yu et al., 2013). This finding is particularly interesting because it links a developmentally associated pathology with the levels of glycine. Complementarily, deprivation of taurine during pregnancy leads to abnormal cortical development in kittens (Sturman, 1988, 1991). Other aspects regarding the physiological role of glycine and taurine have recently been reviewed by Le-Corronc et al. (2011).

Although the role of GlyRs activation during early stages of brain development is yet unclear, there is increasing evidence to hypothesize that GlyRs could be involved in a multitude of cellular processes during development. Depending on that, there are multiple neurodevelopmental associated pathologies in which this receptor could play a role. Mutations of GlyRs are mostly present in the adult GlyR alpha 1 gene, which is almost completely absent during development. However, it has recently been found that a single mutation in the GlyR alpha 2 gene may lead to autism (Piton et al., 2011). This is a missense mutation where an arginine residue is replaced by a leucine (R350L) in the intracellular loop. Although this mutation was found in a female patient, where there are two copies of the gene, one on each X chromosome, the change on the receptor might have a consequence over particular aspects of her disorder. In the case of autism and schizophrenia, another disorder with a developmental component, it has been suggested that the interaction between multiple gene variants rather than one individual mutation is the most likely scenario that originates the disease. Moreover, it has recently been found that GlyR alpha 2 gene is enriched in serotoninergic neurons, which are known to be involved in autism and related disorders (Dougherty et al., 2013). Thus, GlyR alpha 2 gene has been proposed as a strong candidate for further screening in autistic patients (Piton et al., 2011). Along this line, recent findings that describe a role of GlyRs in controlling interneurons migration during embryonic brain development offer a frame to investigate GlyRs on interneuron related disorders (Avila et al., 2013). GABAergic transmission and the appropriate development of cortical interneurons contribute to the right functioning of cortical circuits (Seybold et al., 2012), and defects on these processes are part of the pathophysiology of schizophrenia, autism, and epilepsy (Cobos et al., 2005; Gogolla et al., 2009; Chattopadhyaya and Cristo, 2012; Marin, 2012). It is possible therefore that GlyRs can play a role in these disorders. A more in depth analysis of GlyR alpha 2 KO animals is necessary to address this possibility. In addition, the affinity of GlyRs for glycine or taurine can be markedly increased by alternative splicing or RNA editing in temporal lobe epilepsy (Eichler et al., 2008; Legendre et al., 2009). This is believed to be dependent on the time course of the disease and to affect its evolution. Thus, future research should clarify the role of these variants during development and in neurodevelopmental disorders.

## CONCLUSIONS

**FIGURE 1:**
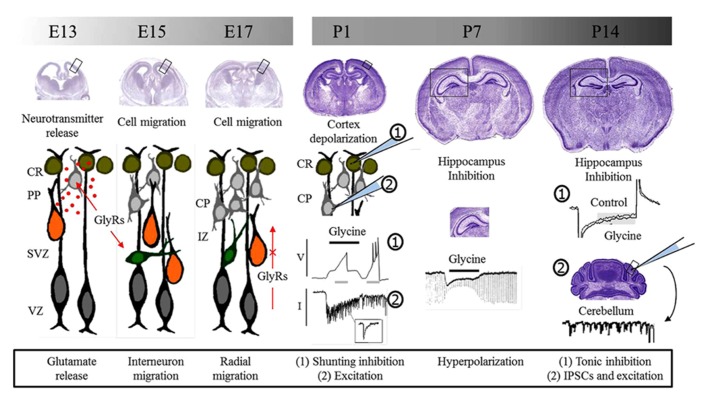
**Glycine receptors and brain development.** Glycine receptors control different processes during pre and postnatal development of the brain. During embryonic development, studies suggest that GlyRs are involved in neurotransmitter release and the control of cell migration. Migrating interneurons are schematized in green while radially migrating cells are shown in orange. Postnatal functions depend on the depolarizing or hyperpolarizing action downstream GlyRs activation. Initially, GlyRs mediate excitation and promote neurotransmitter release and synaptic communication. Later, GlyRs act as inhibitory and take part in inhibitory synapses. E, embryonic day; P, postnatal day, CR, Cajal–Retzius cells; PP, pre-plate; CP, cortical plate; SVZ, sub-ventricular zone; VZ, ventricular zone; IZ, intermediate zone; GlyRs, glycine receptors; V, voltage; I, current; IPSCs, inhibitory postsynaptic currents.

Glycine receptors can be found prominently expressed in the brain and especially during early stages of development. Their expression has been reported since early embryonic stages where they may contribute to different processes (**Figure 1**). Remarkably, recent studies have presented conclusive evidence supporting a role in the control of cell migration. This role seems to be complementary to the activation of GABA receptors that have long been known to contribute in this process. After birth, GlyRs can be found in different structures of the brain displaying a regional differential expression. Although their role in these structures remains unclear, evidence suggest that they may be part of the developmental process in the cortex, hippocampus, cerebellum, and the thalamus. Nevertheless, there is sufficient evidence to consider GlyRs as an important factor controlling neurotransmitter release during postnatal brain development. Consequently, GlyRs may influence the generation of early patterns of activity and the structure of brain circuits. Pathological consequences derived from the malfunction of GlyRs during development are yet to be clarified, but these channels are promising candidates for future research that might offer explanation to rare or complex syndromes that undermine human brain function since early stages of development.

## Conflict of Interest Statement

The authors declare that the research was conducted in the absence of any commercial or financial relationships that could be construed as a potential conflict of interest.

## References

[B1] Agmon A O’Dowd D. K. (1992). NMDA receptor-mediated currents are prominent in the thalamocortical synaptic response before maturation of inhibition. *J. Neurophysiol.* 68 345–349 1381421 10.1152/jn.1992.68.1.345

[B2] Aroeira R. I. Ribeiro J. A. Sebastiao A. M. Valente C. A. (2011). Age-related changes of glycine receptor at the rat hippocampus: from the embryo to the adult. *J. Neurochem.* 118 339–353 10.1111/j.1471-4159.2011.07197.x 21272003

[B3] Avila A. Vidal P. M. Dear T. N. Harvey R. J. Rigo J. M. Nguyen L. (2013). Glycine receptor alpha2 subunit activation promotes cortical interneuron migration. *Cell Rep.* 4 738–750 10.1016/j.celrep.2013.07.016 23954789 PMC3763372

[B4] Backus K. H. Deitmer J. W. Friauf E. (1998). Glycine-activated currents are changed by coincident membrane depolarization in developing rat auditory brainstem neurones. *J. Physiol. *507(Pt 3) 783–794 10.1111/j.1469-7793.1998.783bs.x PMC2230818 9508839

[B5] Ben-Ari Y. (2002). Excitatory actions of GABA during development: the nature of the nurture. *Nat. Rev. Neurosci.* 3 728–739 10.1038/nrn920 12209121

[B6] Ben-Ari Y. Cherubini E. Corradetti R. Gaiarsa J. L. (1989). Giant synaptic potentials in immature rat CA3 hippocampal neurones. *J. Physiol.* 416 303–325 2575165 10.1113/jphysiol.1989.sp017762 PMC1189216

[B7] Ben-Ari Y. Gaiarsa J. L. Tyzio R. Khazipov R. (2007). GABA: a pioneer transmitter that excites immature neurons and generates primitive oscillations. *Physiol. Rev.* 87 1215–1284 10.1152/physrev.00017.2006 17928584

[B8] Ben-Ari Y. Khalilov I. Represa A. Gozlan H. (2004). Interneurons set the tune of developing networks. *Trends Neurosci.* 27 422–427 10.1016/j.tins.2004.05.002 15219742

[B9] Benitez-Diaz P. Miranda-Contreras L. Mendoza-Briceno R. V. Pena-Contreras Z. Palacios-Pru E. (2003). Prenatal and postnatal contents of amino acid neurotransmitters in mouse parietal cortex. *Dev. Neurosci.* 25 366–374 10.1159/000073514 14614264

[B10] Bortone D. Polleux F. (2009). KCC2 expression promotes the termination of cortical interneuron migration in a voltage-sensitive calcium-dependent manner. *Neuron* 62 53–71 10.1016/j.neuron.2009.01.034 19376067 PMC3314167

[B11] Brackmann M. Zhao C. Schmieden V. Braunewell K. H. (2004). Cellular and subcellular localization of the inhibitory glycine receptor in hippocampal neurons. *Biochem. Biophys. Res. Commun.* 324 1137–1142 10.1016/j.bbrc.2004.09.172 15485673

[B12] Breustedt J. Schmitz D. Heinemann U. Schmieden V. (2004). Characterization of the inhibitory glycine receptor on entorhinal cortex neurons. *Eur. J. Neurosci.* 19 1987–1991 10.1111/j.1460-9568.2004.03266.x 15078573

[B13] Brickley S. G. Cull-Candy S. G. Farrant M. (1996). Development of a tonic form of synaptic inhibition in rat cerebellar granule cells resulting from persistent activation of GABAA receptors. *J. Physiol.* 497 753–759 9003560 10.1113/jphysiol.1996.sp021806 PMC1160971

[B14] Caronia-Brown G. Grove E. A. (2011). Timing of cortical interneuron migration is influenced by the cortical hem. *Cereb. Cortex* 21 748–755 10.1093/cercor/bhq142 20713502 PMC3059882

[B15] Chattipakorn S. C. McMahon L. L. (2002). Pharmacological characterization of glycine-gated chloride currents recorded in rat hippocampal slices. *J. Neurophysiol.* 87 1515–1525 11877523 10.1152/jn.00365.2001

[B16] Chattopadhyaya B. Cristo G. D. (2012). GABAergic circuit dysfunctions in neurodevelopmental disorders. *Front. Psychiatry * 3:51 10.3389/fpsyt.2012.00051 PMC3364508 22666213

[B17] Chen R. Q. Wang S. H. Yao W. Wang J. J. Ji F. Yan J. Z. (2011). Role of glycine receptors in glycine-induced LTD in hippocampal CA1 pyramidal neurons. *Neuropsychopharmacology* 36 1948–1958 10.1038/npp.2011.86 21593734 PMC3154115

[B18] Cobos I. Calcagnotto M. E. Vilaythong A. J. Thwin M. T. Noebels J. L. Baraban S. C. (2005). Mice lacking Dlx1 show subtype-specific loss of interneurons, reduced inhibition and epilepsy. *Nat. Neurosci.* 8 1059–1068 10.1038/nn1499 16007083

[B19] Colin I. Rostaing P. Augustin A. Triller A. (1998). Localization of components of glycinergic synapses during rat spinal cord development. *J. Comp. Neurol.* 398 359–372 10.1002/(SICI)1096-9861(19980831)398:3<359::AID-CNE5>3.0.CO;2-Z 9714149

[B20] Cossart R. (2011). The maturation of cortical interneuron diversity: how multiple developmental journeys shape the emergence of proper network function? *Curr.Opin. Neurobiol.* 21 160–168 10.1016/j.conb.2010.10.003 21074988

[B21] Crook J. Hendrickson A. Robinson F. R. (2006). Co-localization of glycine and gaba immunoreactivity in interneurons in Macaca monkey cerebellar cortex. *Neuroscience* 141 1951–1959 10.1016/j.neuroscience.2006.05.012 16784818

[B22] Cubelos B. Gimenez C. Zafra F. (2005). Localization of the GLYT1 glycine transporter at glutamatergic synapses in the rat brain. *Cereb. Cortex* 15 448–459 10.1093/cercor/bhh147 15749988

[B23] Danglot L. Rostaing P. Triller A. Bessis A. (2004). Morphologically identified glycinergic synapses in the hippocampus. *Mol. Cell. Neurosci.* 27 394–403 10.1016/j.mcn.2004.05.007 15555918

[B24] De Saint Jan D. David-Watine B. Korn H. Bregestovski P. (2001). Activation of human alpha1 and alpha2 homomeric glycine receptors by taurine and GABA. *J. Physiol.* 535 741–755 10.1111/j.1469-7793.2001.t01-1-00741.x 11559772 PMC2278820

[B25] Denter D. G. Heck N. Riedemann T. White R. Kilb W. Luhmann H. J. (2010). GABAC receptors are functionally expressed in the intermediate zone and regulate radial migration in the embryonic mouse neocortex. *Neuroscience* 167 124–134 10.1016/j.neuroscience.2010.01.049 20123002

[B26] Dieudonne S. (1995). Glycinergic synaptic currents in Golgi cells of the rat cerebellum. *Proc. Natl. Acad. Sci. U.S.A.* 92 1441–1445 10.1073/pnas.92.5.1441 7877998 PMC42535

[B27] Dougherty J. D. Maloney S. E. Wozniak D. F. Rieger M. A. Sonnenblick L. Coppola G. (2013). The disruption of Celf6, a gene identified by translational profiling of serotonergic neurons, results in autism-related behaviors. *J. Neurosci.* 33 2732–2753 10.1523/JNEUROSCI.4762-12.2013 23407934 PMC3711589

[B28] Dugue G. P. Dumoulin A. Triller A. Dieudonne S. (2005). Target-dependent use of co-released inhibitory transmitters at central synapses. *J. Neurosci.* 25 6490–6498 10.1523/JNEUROSCI.1500-05.2005 16014710 PMC6725433

[B29] Dumoulin A. Triller A. Dieudonne S. (2001). IPSC kinetics at identified GABAergic and mixed GABAergic and glycinergic synapses onto cerebellar Golgi cells. *J. Neurosci.* 21 6045–6057 11487628 10.1523/JNEUROSCI.21-16-06045.2001 PMC6763194

[B30] Dunning D. D. Hoover C. L. Soltesz I. Smith M. A O’Dowd D. K. (1999). GABA(A) receptor-mediated miniature postsynaptic currents and alpha-subunit expression in developing cortical neurons. *J. Neurophysiol.* 82 3286–3297 10601460 10.1152/jn.1999.82.6.3286

[B31] Dupont E. Hanganu I. L. Kilb W. Hirsch S. Luhmann H. J. (2006). Rapid developmental switch in the mechanisms driving early cortical columnar networks. *Nature* 439 79–83 10.1038/nature04264 16327778

[B32] Ehrlich I. Lohrke S. Friauf E. (1999). Shift from depolarizing to hyperpolarizing glycine action in rat auditory neurones is due to age-dependent Cl^-^ regulation. *J. Physiol.* 520 121–137 10.1111/j.1469-7793.1999.00121.x 10517806 PMC2269580

[B33] Eichler S. A. Forstera B. Smolinsky B. Juttner R. Lehmann T. N. Fahling M. (2009). Splice-specific roles of glycine receptor alpha3 in the hippocampus. *Eur. J. Neurosci.* 30 1077–1091 10.1111/j.1460-9568.2009.06903.x 19723286

[B34] Eichler S. A. Kirischuk S. Juttner R. Schaefermeier P. K. Legendre P. Lehmann T. N. (2008). Glycinergic tonic inhibition of hippocampal neurons with depolarizing GABAergic transmission elicits histopathological signs of temporal lobe epilepsy. *J. Cell Mol. Med.* 12 2848–2866 10.1111/j.1582-4934.2008.00357.x 19210758 PMC3828897

[B35] Eulenburg V. Retiounskaia M. Papadopoulos T. Gomeza J. Betz H. (2010). Glial glycine transporter 1 function is essential for early postnatal survival but dispensable in adult mice. *Glia* 58 1066–1073 10.1002/glia.20987 20468048

[B36] Flint A. C. Liu X. Kriegstein A. R. (1998). Nonsynaptic glycine receptor activation during early neocortical development. *Neuron* 20 43–53 10.1016/S0896-6273(00)80433-X 9459441

[B37] Frostholm A. Rotter A. (1985). Glycine receptor distribution in mouse CNS: autoradiographic localization of [3H]strychnine binding sites. *Brain Res. Bull.* 15 473–486 10.1016/0361-9230(85)90038-3 2998565

[B38] Gao B. X. Stricker C. Ziskind-Conhaim L. (2001). Transition from GABAergic to glycinergic synaptic transmission in newly formed spinal networks. *J. Neurophysiol.* 86 492–502 11431527 10.1152/jn.2001.86.1.492

[B39] Ghavanini A. A. Mathers D. A. Puil E. (2005). Glycinergic inhibition in thalamus revealed by synaptic receptor blockade. *Neuropharmacology* 49 338–349 10.1016/j.neuropharm.2005.03.013 15993440

[B40] Gogolla N. Leblanc J. J. Quast K. B. Sudhof T. C. Fagiolini M. Hensch T. K. (2009). Common circuit defect of excitatory–inhibitory balance in mouse models of autism. *J. Neurodev. Disord.* 1 172–181 10.1007/s11689-009-9023-x 20664807 PMC2906812

[B41] Gomeza J. Ohno K. Hulsmann S. Armsen W. Eulenburg V. Richter D. W. (2003). Deletion of the mouse glycine transporter 2 results in a hyperekplexia phenotype and postnatal lethality. *Neuron* 40 797–806 10.1016/S0896-6273(03)00673-1 14622583

[B42] Gozlan H. Ben-Ari Y. (2003). Interneurons are the source and the targets of the first synapses formed in the rat developing hippocampal circuit. *Cereb. Cortex* 13 684–692 10.1093/cercor/13.6.684 12764045

[B43] Gutierrez R. (2003). The GABAergic phenotype of the “glutamatergic” granule cells of the dentate gyrus. *Prog. Neurobiol.* 71 337–358 10.1016/j.pneurobio.2003.11.004 14757115

[B44] Harvey R. J. Depner U. B. Wassle H. Ahmadi S. Heindl C. Reinold H. (2004). GlyR alpha3: an essential target for spinal PGE2-mediated inflammatory pain sensitization. *Science* 304 884–887 10.1126/science.1094925 15131310

[B45] Heng J. I. Moonen G. Nguyen L. (2007). Neurotransmitters regulate cell migration in the telencephalon. *Eur. J. Neurosci.* 26 537–546 10.1111/j.1460-9568.2007.05694.x 17686035

[B46] Hennou S. Khalilov I. Diabira D. Ben-Ari Y. Gozlan H. (2002). Early sequential formation of functional GABA(A) and glutamatergic synapses on CA1 interneurons of the rat foetal hippocampus. *Eur. J. Neurosci.* 16 197–208 10.1046/j.1460-9568.2002.02073.x 12169102

[B47] Hussy N. Deleuze C. Pantaloni A. Desarmenien M. G. Moos F. (1997). Agonist action of taurine on glycine receptors in rat supraoptic magnocellular neurones: possible role in osmoregulation. *J. Physiol.* 502 609–621 10.1111/j.1469-7793.1997.609bj.x 9279812 PMC1159532

[B48] Ichinohe A. Kure S. Mikawa S. Ueki T. Kojima K. Fujiwara K. (2004). Glycine cleavage system in neurogenic regions. *Eur. J. Neurosci.* 19 2365–2370 10.1111/j.0953-816X.2004.03345.x 15128390

[B49] Inoue K. Furukawa T. Kumada T. Yamada J. Wang T. Inoue R. (2012). Taurine inhibits K^+^–Cl^-^ cotransporter KCC2 to regulate embryonic Cl^-^ homeostasis via with-no-lysine (WNK) protein kinase signaling pathway. *J. Biol. Chem.* 287 20839–20850 10.1074/jbc.M111.319418 22544747 PMC3375508

[B50] Ito S. Cherubini E. (1991). Strychnine-sensitive glycine responses of neonatal rat hippocampal neurones. *J. Physiol.* 440 67–83 1804982 10.1113/jphysiol.1991.sp018696 PMC1180140

[B51] Jonsson S. Morud J. Pickering C. Adermark L. Ericson M. Soderpalm B. (2012). Changes in glycine receptor subunit expression in forebrain regions of the Wistar rat over development. *Brain Res.* 1446 12–21 10.1016/j.brainres.2012.01.050 22330726

[B52] Jursky F. Nelson N. (1996). Developmental expression of the glycine transporters GLYT1 and GLYT2 in mouse brain. *J. Neurochem.* 67 336–344 10.1046/j.1471-4159.1996.67010336.x 8667011

[B53] Jursky F. Tamura S. Tamura A. Mandiyan S. Nelson H. Nelson N. (1994). Structure, function and brain localization of neurotransmitter transporters. *J. Exp. Biol.* 196 283–295 7823028 10.1242/jeb.196.1.283

[B54] Kaneda M. Farrant M. Cull-Candy S. G. (1995). Whole-cell and single-channel currents activated by GABA and glycine in granule cells of the rat cerebellum. *J. Physiol. *485(Pt 2) 419–435 10.1113/jphysiol.1995.sp020739 PMC1158002 7545231

[B55] Kawa K. (2003a). Glycine facilitates transmitter release at developing synapses: a patch clamp study from Purkinje neurons of the newborn rat. *Brain Res. Dev. Brain Res.* 144 57–71 10.1016/S0165-3806(03)00159-7 12888217

[B56] Kawa K. (2003b). Glycine receptors and glycinergic synaptic transmission in the deep cerebellar nuclei of the rat: a patch-clamp study. *J. Neurophysiol.* 90 3490–3500 10.1152/jn.00447.2003 12867529

[B57] Keck T. Lillis K. P. Zhou Y. D. White J. A. (2008). Frequency-dependent glycinergic inhibition modulates plasticity in hippocampus. *J. Neurosci.* 28 7359–7369 10.1523/JNEUROSCI.5618-07.2008 18632940 PMC2577594

[B58] Kilb W. Hanganu I. L. Okabe A. Sava B. A. Shimizu-Okabe C. Fukuda A. (2008). Glycine receptors mediate excitation of subplate neurons in neonatal rat cerebral cortex. *J. Neurophysiol.* 100 698–707 10.1152/jn.00657.2007 18562558

[B59] Kilb W. Ikeda M. Uchida K. Okabe A. Fukuda A. Luhmann H. J. (2002). Depolarizing glycine responses in Cajal–Retzius cells of neonatal rat cerebral cortex. *Neuroscience* 112 299–307 10.1016/S0306-4522(02)00071-4 12044448

[B60] Kirsch J. Kuhse J. Betz H. (1995). Targeting of glycine receptor subunits to gephyrin-rich domains in transfected human embryonic kidney cells. *Mol. Cell. Neurosci.* 6 450–461 10.1006/mcne.1995.1033 8581315

[B61] Konur S. Ghosh A. (2005). Calcium signaling and the control of dendritic development. *Neuron* 46 401–405 10.1016/j.neuron.2005.04.022 15882639

[B62] Kotak V. C. Korada S. Schwartz I. R. Sanes D. H. (1998). A developmental shift from GABAergic to glycinergic transmission in the central auditory system. *J. Neurosci.* 18 4646–4655 9614239 10.1523/JNEUROSCI.18-12-04646.1998 PMC6792682

[B63] Kubota H. Alle H. Betz H. Geiger J. R. (2010). Presynaptic glycine receptors on hippocampal mossy fibers. *Biochem. Biophys. Res. Commun.* 393 587–591 10.1016/j.bbrc.2010.02.019 20152805

[B64] Kuhse J. Kuryatov A. Maulet Y. Malosio M. L. Schmieden V. Betz H. (1991). Alternative splicing generates two isoforms of the alpha 2 subunit of the inhibitory glycine receptor. *FEBS Lett.* 283 73–77 10.1016/0014-5793(91)80557-J 1645300

[B65] Kunz P. A. Burette A. C. Weinberg R. J. Philpot B. D. (2012). Glycine receptors support excitatory neurotransmitter release in developing mouse visual cortex. *J. Physiol.* 590 5749–5764 10.1113/jphysiol.2012.241299 22988142 PMC3528989

[B66] Lall D. Armbruster A. Ruffert K. Betz H. Eulenburg V. (2012). Transport activities and expression patterns of glycine transporters 1 and 2 in the developing murine brain stem and spinal cord. *Biochem. Biophys. Res. Commun.* 423 661–666 10.1016/j.bbrc.2012.06.007 22695116

[B67] Le-Corronc H. Rigo J. M. Branchereau P. Legendre P. (2011). GABA(A) receptor and glycine receptor activation by paracrine/autocrine release of endogenous agonists: more than a simple communication pathway. *Mol. Neurobiol.* 44 28–52 10.1007/s12035-011-8185-1 21547557

[B68] Lee E. A. Cho J. H. Choi I. S. Nakamura M. Park H. M. Lee J. J. (2009). Presynaptic glycine receptors facilitate spontaneous glutamate release onto hilar neurons in the rat hippocampus. *J. Neurochem.* 109 275–286 10.1111/j.1471-4159.2009.05960.x 19200346

[B69] Lee H. Chen C. X. Liu Y. J. Aizenman E. Kandler K. (2005). KCC2 expression in immature rat cortical neurons is sufficient to switch the polarity of GABA responses. *Eur. J. Neurosci.* 21 2593–2599 10.1111/j.1460-9568.2005.04084.x 15932617 PMC2945502

[B70] Legendre P. Forstera B. Juttner R. Meier J. C. (2009). Glycine receptors caught between genome and proteome-functional implications of RNA editing and splicing. *Front. Mol. Neurosci. *2:23. 10.3389/neuro.02.023.2009 PMC2779093 19936314

[B71] Li Y. Xu T. L. (2002). State-dependent cross-inhibition between anionic GABA(A) and glycine ionotropic receptors in rat hippocampal CA1 neurons. *Neuroreport* 13 223–226 10.1097/00001756-200202110-00010 11893914

[B72] LoTurco J. J. Owens D. F. Heath M. J. Davis M. B. Kriegstein A. R. (1995). GABA and glutamate depolarize cortical progenitor cells and inhibit DNA synthesis. *Neuron* 15 1287–1298 10.1016/0896-6273(95)90008-X 8845153

[B73] Lu T. Rubio M. E. Trussell L. O. (2008). Glycinergic transmission shaped by the corelease of GABA in a mammalian auditory synapse. *Neuron* 57 524–535 10.1016/j.neuron.2007.12.010 18304482

[B74] Lynch J. W. (2009). Native glycine receptor subtypes and their physiological roles. *Neuropharmacology* 56 303–309 10.1016/j.neuropharm.2008.07.034 18721822

[B75] Lynch J. W. Callister R. J. (2006). Glycine receptors: a new therapeutic target in pain pathways. *Curr. Opin. Invest. Drugs* 7 48–53 16425671

[B76] Malosio M. L. Grenningloh G. Kuhse J. Schmieden V. Schmitt B. Prior P. (1991a). Alternative splicing generates two variants of the alpha 1 subunit of the inhibitory glycine receptor. *J. Biol. Chem.* 266 2048–2053 1703526

[B77] Malosio M. L. Marqueze-Pouey B. Kuhse J. Betz H. (1991b). Widespread expression of glycine receptor subunit mRNAs in the adult and developing rat brain. *EMBO J.* 10 2401–2409 1651228 10.1002/j.1460-2075.1991.tb07779.x PMC452935

[B78] Manent J. B. Demarque M. Jorquera I. Pellegrino C. Ben-Ari Y. Aniksztejn L. (2005). A noncanonical release of GABA and glutamate modulates neuronal migration. *J. Neurosci.* 25 4755–4765 10.1523/JNEUROSCI.0553-05.2005 15888651 PMC6724769

[B79] Manent J. B. Represa A. (2007). Neurotransmitters and brain maturation: early paracrine actions of GABA and glutamate modulate neuronal migration. *Neuroscientist* 13 268–279 10.1177/1073858406298918 17519369

[B80] Marin O. (2012). Interneuron dysfunction in psychiatric disorders. *Nat. Rev. Neurosci.* 13 107–120 22251963 10.1038/nrn3155

[B81] Martina M. Gorfinkel Y. Halman S. Lowe J. A. Periyalwar P. Schmidt C. J. (2004). Glycine transporter type 1 blockade changes NMDA receptor-mediated responses and LTP in hippocampal CA1 pyramidal cells by altering extracellular glycine levels. *J. Physiol.* 557 489–500 10.1113/jphysiol.2004.063321 15064326 PMC1665089

[B82] Marty S. Berninger B. Carroll P. Thoenen H. (1996). GABAergic stimulation regulates the phenotype of hippocampal interneurons through the regulation of brain-derived neurotrophic factor. *Neuron* 16 565–570 10.1016/S0896-6273(00)80075-6 8785053

[B83] Matzenbach B. Maulet Y. Sefton L. Courtier B. Avner P. Guenet J. L. (1994). Structural analysis of mouse glycine receptor alpha subunit genes. Identification and chromosomal localization of a novel variant. *J. Biol. Chem.* 269 2607–2612 7507926

[B84] McDearmid J. R. Liao M. Drapeau P. (2006). Glycine receptors regulate interneuron differentiation during spinal network development. *Proc. Natl. Acad. Sci. U.S.A.* 103 9679–9684 10.1073/pnas.0504871103 16763051 PMC1480466

[B85] Moss S. J. Smart T. G. (2001). Constructing inhibitory synapses. *Nat. Rev. Neurosci.* 2 240–250 10.1038/35067500 11283747

[B86] Muller E. Le Corronc H. Scain A. L. Triller A. Legendre P. (2008). Despite GABAergic neurotransmission, GABAergic innervation does not compensate for the defect in glycine receptor postsynaptic aggregation in spastic mice. *Eur. J. Neurosci.* 27 2529–2541 10.1111/j.1460-9568.2008.06217.x 18445051

[B87] Muller E. Le Corronc H. Triller A. Legendre P. (2006). Developmental dissociation of presynaptic inhibitory neurotransmitter and postsynaptic receptor clustering in the hypoglossal nucleus. *Mol. Cell. Neurosci.* 32 254–273 10.1016/j.mcn.2006.04.007 16765056

[B88] Nabekura J. Katsurabayashi S. Kakazu Y. Shibata S. Matsubara A. Jinno S. (2004). Developmental switch from GABA to glycine release in single central synaptic terminals. *Nat. Neurosci.* 7 17–23 10.1038/nn1170 14699415

[B89] Nguyen L. Malgrange B. Belachew S. Rogister B. Rocher V. Moonen G. (2002). Functional glycine receptors are expressed by postnatal nestin-positive neural stem/progenitor cells. *Eur. J. Neurosci.* 15 1299–1305 10.1046/j.1460-9568.2002.01966.x 11994124

[B90] Nguyen L. Rigo J. M. Rocher V. Belachew S. Malgrange B. Rogister B. (2001). Neurotransmitters as early signals for central nervous system development. *Cell Tissue Res.* 305 187–202 10.1007/s004410000343 11545256

[B91] Nikolic Z. Laube B. Weber R. G. Lichter P. Kioschis P. Poustka A. (1998). The human glycine receptor subunit alpha3. *Glra*3 gene structure, chromosomal localization, and functional characterization of alternative transcripts. *J. Biol. Chem.* 273 19708–19714 10.1074/jbc.273.31.19708 9677400

[B92] Nimmervoll B. Denter D. G. Sava I. Kilb W. Luhmann H. J. (2011). Glycine receptors influence radial migration in the embryonic mouse neocortex. *Neuroreport* 22 509–513 10.1097/WNR.0b013e328348aafe 21666516

[B93] Noctor S. C. Martinez-Cerdeno V. Ivic L. Kriegstein A. R. (2004). Cortical neurons arise in symmetric and asymmetric division zones and migrate through specific phases. *Nat. Neurosci.* 7 136–144 10.1038/nn1172 14703572

[B94] Oertel J. Villmann C. Kettenmann H. Kirchhoff F. Becker C. M. (2007). A novel glycine receptor beta subunit splice variant predicts an unorthodox transmembrane topology. Assembly into heteromeric receptor complexes*. J. Biol. Chem.* 282 2798–2807 10.1074/jbc.M608941200 17145751

[B95] Okabe A. Kilb W. Shimizu-Okabe C. Hanganu I. L. Fukuda A. Luhmann H. J. (2004). Homogenous glycine receptor expression in cortical plate neurons and Cajal–Retzius cells of neonatal rat cerebral cortex. *Neuroscience* 123 715–724 10.1016/j.neuroscience.2003.10.014 14706783

[B96] Owens D. F. Boyce L. H. Davis M. B. Kriegstein A. R. (1996). Excitatory GABA responses in embryonic and neonatal cortical slices demonstrated by gramicidin perforated-patch recordings and calcium imaging. *J. Neurosci.* 16 6414–6423 8815920 10.1523/JNEUROSCI.16-20-06414.1996 PMC6578913

[B97] Pedroarena C. M. Kamphausen S. (2008). Glycinergic synaptic currents in the deep cerebellar nuclei. *Neuropharmacology* 54 784–795 10.1016/j.neuropharm.2007.12.005 18234240

[B98] Piton A. Gauthier J. Hamdan F. F. Lafreniere R. G. Yang Y. Henrion E. (2011). Systematic resequencing of X-chromosome synaptic genes in autism spectrum disorder and schizophrenia. *Mol. Psychiatry* 16 867–880 10.1038/mp.2010.54 20479760 PMC3289139

[B99] Pla R. Borrell V. Flames N. Marin O. (2006). Layer acquisition by cortical GABAergic interneurons is independent of Reelin signaling. *J. Neurosci.* 26 6924–6934 10.1523/JNEUROSCI.0245-06.2006 16807322 PMC6673924

[B100] Platel J. C. Boisseau S. Dupuis A. Brocard J. Poupard A. Savasta M. (2005). Na^+^ channel-mediated Ca^2^^+^ entry leads to glutamate secretion in mouse neocortical preplate. *Proc. Natl. Acad. Sci. U.S.A.* 102 19174–19179 10.1073/pnas.0504540102 16357207 PMC1323152

[B101] Pribilla I. Takagi T. Langosch D. Bormann J. Betz H. (1992). The atypical M2 segment of the beta subunit confers picrotoxinin resistance to inhibitory glycine receptor channels. *EMBO J.* 11 4305–4311 1385113 10.1002/j.1460-2075.1992.tb05529.x PMC557003

[B102] Probst A. Cortes R. Palacios J. M. (1986). The distribution of glycine receptors in the human brain. A light microscopic autoradiographic study using [3H]strychnine. *Neuroscience* 17 11–35 10.1016/0306-4522(86)90222-8 3008022

[B103] Rahman J. Latal A. T. Besser S. Hirrlinger J. Hulsmann S. (2013). Mixed miniature postsynaptic currents resulting from co-release of glycine and GABA recorded from glycinergic neurons in the neonatal respiratory network. *Eur. J. Neurosci.* 37 1229–1241 10.1111/ejn.12136 23347272

[B104] Rees M. I. Harvey K. Ward H. White J. H. Evans L. Duguid I. C. (2003). Isoform heterogeneity of the human gephyrin gene (GPHN), binding domains to the glycine receptor, and mutation analysis in hyperekplexia. *J. Biol. Chem.* 278 24688–24696 10.1074/jbc.M301070200 12684523

[B105] Represa A. Ben-Ari Y. (2005). Trophic actions of GABA on neuronal development. *Trends Neurosci.* 28 278–283 10.1016/j.tins.2005.03.010 15927682

[B106] Ripps H. Shen W. (2012). Review: taurine: a “very essential” amino acid. *Mol. Vis.* 18 2673–2686 23170060 PMC3501277

[B107] Sahara S. Yanagawa Y. O’Leary D. D. Stevens C. F. (2012). The fraction of cortical GABAergic neurons is constant from near the start of cortical neurogenesis to adulthood. *J. Neurosci.* 32 4755–4761 10.1523/JNEUROSCI.6412-11.2012 22492031 PMC3325497

[B108] Sauer J. F. Bartos M. (2011). Postnatal differentiation of cortical interneuron signalling. *Eur. J. Neurosci.* 34 1687–1696 10.1111/j.1460-9568.2011.07872.x 22103425

[B109] Scain A. L. Le Corronc H. Allain A. E. Muller E. Rigo J. M. Meyrand P. (2010). Glycine release from radial cells modulates the spontaneous activity and its propagation during early spinal cord development. *J. Neurosci.* 30 390–403 10.1523/JNEUROSCI.2115-09.2010 20053920 PMC6632542

[B110] Schmieden V. Kuhse J. Betz H. (1992). Agonist pharmacology of neonatal and adult glycine receptor alpha subunits: identification of amino acid residues involved in taurine activation. *EMBO J.* 11 2025–2032 1376243 10.1002/j.1460-2075.1992.tb05259.x PMC556667

[B111] Seybold B. A. Stanco A. Cho K. K. Potter G. B. Kim C. Sohal V. S. (2012). Chronic reduction in inhibition reduces receptive field size in mouse auditory cortex. *Proc. Natl. Acad. Sci. U.S.A.* 109 13829–13834 10.1073/pnas.1205909109 22753490 PMC3427089

[B112] Singer J. H. (2008). GABA is an endogenous ligand for synaptic glycine receptors. *Neuron* 57 475–477 10.1016/j.neuron.2008.02.007 18304476

[B113] Singer J. H. Talley E. M. Bayliss D. A. Berger A. J. (1998). Development of glycinergic synaptic transmission to rat brain stem motoneurons. *J. Neurophysiol.* 80 2608–2620 9819267 10.1152/jn.1998.80.5.2608

[B114] Song W. Chattipakorn S. C. McMahon L. L. (2006). Glycine-gated chloride channels depress synaptic transmission in rat hippocampus. *J. Neurophysiol.* 95 2366–2379 10.1152/jn.00386.2005 16381810

[B115] Sorensen S. A. Bernard A. Menon V. Royall J. J. Glattfelder K. J. Hirokawa K. (2013). Correlated gene expression and target specificity demonstrate excitatory projection neuron diversity. *Cereb. Cortex*. 10.1093/cercor/bht243 [Epub ahead of print] 24014670

[B116] Soria J. M. Valdeolmillos M. (2002). Receptor-activated calcium signals in tangentially migrating cortical cells. *Cereb. Cortex* 12 831–839 10.1093/cercor/12.8.831 12122031

[B117] Sturman J. A. (1988). Taurine in development. *J. Nutr.* 118 1169–1176 3054019 10.1093/jn/118.10.1169

[B118] Sturman J. A. (1991). Dietary taurine and feline reproduction and development. *J. Nutr.* 121 S166–S170 1941217 10.1093/jn/121.suppl_11.S166

[B119] Thio L. L. Shanmugam A. Isenberg K. Yamada K. (2003). Benzodiazepines block alpha2-containing inhibitory glycine receptors in embryonic mouse hippocampal neurons. *J. Neurophysiol.* 90 89–99 10.1152/jn.00612.2002 12660352

[B120] Turecek R. Trussell L. O. (2001). Presynaptic glycine receptors enhance transmitter release at a mammalian central synapse. *Nature* 411 587–590 10.1038/35079084 11385573

[B121] Tyzio R. Holmes G. L. Ben-Ari Y. Khazipov R. (2007). Timing of the developmental switch in GABA(A) mediated signaling from excitation to inhibition in CA3 rat hippocampus using gramicidin perforated patch and extracellular recordings. *Epilepsia *48(Suppl. 5) 96–105 10.1111/j.1528-1167.2007.01295.x 17910587

[B122] van den Pol A. N. (2004). Developing neurons make the switch. *Nat. Neurosci.* 7 7–8 10.1038/nn0104-7 14699410

[B123] van den Pol A. N. Gorcs T. (1988). Glycine and glycine receptor immunoreactivity in brain and spinal cord. *J. Neurosci.* 8 472–492 2892900 10.1523/JNEUROSCI.08-02-00472.1988 PMC6569307

[B124] Verheugen J. A. Fricker D. Miles R. (1999). Noninvasive measurements of the membrane potential and GABAergic action in hippocampal interneurons. *J. Neurosci.* 19 2546–2555 10087068 10.1523/JNEUROSCI.19-07-02546.1999 PMC6786065

[B125] Waseem T. V. Fedorovich S. V. (2010). Presynaptic glycine receptors influence plasma membrane potential and glutamate release. *Neurochem. Res.* 35 1188–1195 10.1007/s11064-010-0174-7 20431942

[B126] Watanabe E. Akagi H. (1995). Distribution patterns of mRNAs encoding glycine receptor channels in the developing rat spinal cord. *Neurosci. Res.* 23 377–382 10.1016/0168-0102(95)00972-V 8602277

[B127] Wegner F. Kraft R. Busse K. Hartig W. Ahrens J. Leffler A. (2012). Differentiated human midbrain-derived neural progenitor cells express excitatory strychnine-sensitive glycine receptors containing alpha2beta subunits. *PLoS ONE *7:e36946. 10.1371/journal.pone.0036946 PMC3350492 22606311

[B128] Wu W. L. Ziskind-Conhaim L. Sweet M. A. (1992). Early development of glycine- and GABA-mediated synapses in rat spinal cord. *J. Neurosci.* 12 3935–3945 1403091 10.1523/JNEUROSCI.12-10-03935.1992 PMC6575960

[B129] Xu T. L. Gong N. (2010). Glycine and glycine receptor signaling in hippocampal neurons: diversity, function and regulation. *Prog. Neurobiol.* 91 349–361 10.1016/j.pneurobio.2010.04.008 20438799

[B130] Yee B. K. Balic E. Singer P. Schwerdel C. Grampp T. Gabernet L. (2006). Disruption of glycine transporter 1 restricted to forebrain neurons is associated with a procognitive and antipsychotic phenotypic profile. *J. Neurosci.* 26 3169–3181 10.1523/JNEUROSCI.5120-05.2006 16554468 PMC6674096

[B131] Yoshida M. Fukuda S. Tozuka Y. Miyamoto Y. Hisatsune T. (2004). Developmental shift in bidirectional functions of taurine-sensitive chloride channels during cortical circuit formation in postnatal mouse brain. *J. Neurobiol.* 60 166–175 10.1002/neu.20003 15266648

[B132] Young-Pearse T. L. Ivic L. Kriegstein A. R. Cepko C. L. (2006). Characterization of mice with targeted deletion of glycine receptor alpha 2. *Mol. Cell. Biol.* 26 5728–5734 10.1128/MCB.00237-06 16847326 PMC1592777

[B133] Yu T. W. Chahrour M. H. Coulter M. E. Jiralerspong S. Okamura-Ikeda K. Ataman B. (2013). Using whole-exome sequencing to identify inherited causes of autism. *Neuron* 77 259–273 10.1016/j.neuron.2012.11.002 23352163 PMC3694430

[B134] Zeilhofer H. U. (2005). The glycinergic control of spinal pain processing. *Cell. Mol. Life Sci.* 62 2027–2035 10.1007/s00018-005-5107-2 15968463 PMC11139092

[B135] Zhang L. H. Gong N. Fei D. Xu L. Xu T. L. (2008). Glycine uptake regulates hippocampal network activity via glycine receptor-mediated tonic inhibition. *Neuropsychopharmacology* 33 701–711 10.1038/sj.npp.1301449 17522628

[B136] Zhu L. Lovinger D. Delpire E. (2005). Cortical neurons lacking KCC2 expression show impaired regulation of intracellular chloride. * J. Neurophysiol.* 93 1557–1568 10.1152/jn.00616.2004 15469961

